# Optimal Learning Rules for Discrete Synapses

**DOI:** 10.1371/journal.pcbi.1000230

**Published:** 2008-11-28

**Authors:** Adam B. Barrett, M. C. W. van Rossum

**Affiliations:** Institute for Adaptive and Neural Computation, University of Edinburgh, Edinburgh, United Kingdom; UFR Biomédicale de l'Université René Descartes, France

## Abstract

There is evidence that biological synapses have a limited number of discrete weight states. Memory storage with such synapses behaves quite differently from synapses with unbounded, continuous weights, as old memories are automatically overwritten by new memories. Consequently, there has been substantial discussion about how this affects learning and storage capacity. In this paper, we calculate the storage capacity of discrete, bounded synapses in terms of Shannon information. We use this to optimize the learning rules and investigate how the maximum information capacity depends on the number of synapses, the number of synaptic states, and the coding sparseness. Below a certain critical number of synapses per neuron (comparable to numbers found in biology), we find that storage is similar to unbounded, continuous synapses. Hence, discrete synapses do not necessarily have lower storage capacity.

## Author Summary

It is believed that the neural basis of learning and memory is change in the strength of synaptic connections between neurons. Much theoretical work on this topic assumes that the strength, or weight, of a synapse may vary continuously and be unbounded. More recent studies have considered synapses that have a limited number of discrete states. In dynamical models of such synapses, old memories are automatically overwritten by new memories, and it has been previously difficult to optimize performance using standard capacity measures, for stronger learning typically implies faster forgetting. Here, we propose an information theoretic measure of storage capacity of such forgetting systems, and use this to optimize the learning rules. We find that for parameters comparable to those found in biology, capacity of discrete synapses is similar to that of unbounded, continuous synapses, provided the number of synapses per neuron is limited. Our findings are relevant for experiments investigating the precise nature of synaptic changes during learning, and also pave a path for further work on building biologically realistic memory models.

## Introduction

Memory in biological neural systems is believed to be stored in the synaptic weights. Numerous computational models of such memory systems have been constructed in order to study their properties and to explore potential hardware implementations. Storage capacity and optimal learning rules have been studied both for single-layer associative networks [1],[2], studied here, and for auto-associative networks [3],[4]. Commonly, synaptic weights in such models are represented by unbounded, continuous real numbers.

However, in biology, as well as in potential hardware, synaptic weights should take values between certain bounds. Furthermore, synapses might be restricted to have a limited number of synaptic states, e.g. the synapse might be binary. Although binary synapses might have limited storage capacity, they can be made more robust to biochemical noise than continuous synapses [5]. Consistent with this, experiments suggest that synaptic weight changes occur in steps. For example, putative single synapse experiments show that a switch-like increment or reduction to the excitatory post-synaptic current can be induced by pairing brief pre-synaptic stimulation with appropriate post-synaptic depolarization [6],[7].

Networks with bounded synapses have the palimpsest property, i.e. old memories decay automatically as they are overwritten by new ones [8]–[15]. In contrast, in networks with continuous, unbounded synapses, storing additional memories reduces the quality of recent and old memories equally (see section *Comparison to continuous, unbounded synapses*). Forgetting of old memories must in that case be explicitly incorporated, for instance via a weight decay mechanism [16],[17]. The automatic forgetting of discrete, bounded synapses allows one to study learning in a realistic equilibrium context, in which there can be continual storage of new information.

It is common to use the signal-to-noise ratio (SNR) to quantify memory storage in neural networks [2],[18]. The SNR measures the separation between responses of the network; the higher the SNR, the more the memory stands out and the less likely it will be lost or distorted. When weights are unbounded, each stored pattern has the same SNR. Storage capacity can then be defined as the maximum number of patterns for which the SNR is larger than some fixed, minimum value.

However, for discrete, bounded synapses performance must be characterized by *two* quantities: the initial SNR, and its decay rate. Ideally, a memory has a high SNR and a slow decay, but altering learning rules typically results in either 1) an increase in memory lifetime but a decrease in initial SNR [18], or 2) an increase in initial SNR but a decrease in memory lifetime. Optimization of the learning rule is ambivalent because an arbitrary trade-off must be made between these two effects. In this paper we resolve this conflict between learning and forgetting by analyzing the capacity of synapses in terms of Shannon information. We describe a framework for calculating the information capacity of bounded, discrete synapses, and use this to find optimal learning rules.

We model a single neuron, and investigate how information capacity depends on the number of synapses and the number of synaptic states. We find that below a critical number of synapses, the total capacity is linear in the number of synapses, while for more synapses the capacity grows only as the square root of the number of synapses per neuron. This critical number is dependent on the sparseness of the patterns stored, as well as on the number of synaptic states. Furthermore, when increasing the number of synaptic states, the information initially grows linearly with the number of states, but saturates for many states. Interestingly, for biologically realistic parameters, capacity is just at this critical point, suggesting that the number of synapses per neuron is limited to prevent sub-optimal learning. Finally, the capacity measure allows direct comparison of discrete with continuous synapses, showing that under the right conditions their capacities are comparable.

## Results

### Setup and Definitions

The single neuron learning paradigm we consider is as follows: at each time-step during the learning phase, a binary pattern is presented and the synapses are updated in an unsupervised manner with a stochastic learning rule. High inputs lead to potentiation, and low inputs to depression of the synapses. Note that if we assume that the inputs cause sufficient post-synaptic activity, the learning rule can be thought of as Hebbian: high (low) pre-synaptic activity paired with post-synaptic activity leads to potentiation (depression). After the learning phase, the neuron is tested with both learned and novel patterns, and it has to perform a recognition task and decide which patterns were learned and which are novel. Alternatively, one can do a (supervised) association task in which some patterns have to be associated with a high output, and others with a low output. This gives qualitatively similar results (see *Associative learning* below).

**Figure 1:**
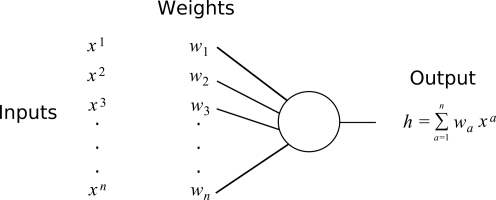
Setup and definitions. Binary input vectors *x^a^* are presented, with each component having probability *p* of being in the high state. Synaptic weights *w_a_* occupy one of *W* discrete states, whose values are equidistantly spaced around zero. The output *h* is the inner product of the vector of inputs with the weight vector.

More precisely, we consider the setup depicted in Figure 1. A neuron has *n* inputs, with weights *w_a_*, *a* = 1,…,*n*. At each time-step it stores a *n*-dimensional binary pattern with independent entries *x^a^*. The probability of a given entry in the pattern being high is given by the sparseness parameter *p*. We set the value of *x* for the low input state equal to −*p*, and the high state to *q* = (1−*p*), so that the probability density for inputs is given by *P*(*x*) = *qδ*(*x*+*p*)+*pδ*(*x*−*q*). Note that 〈*x*〉 = 0. Using the expression for the SNR below, it can be shown that this is optimal, c.f. [2]. We assume that 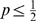, as the case 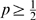 is fully analogous.

Each synapse occupies one of *W* states. The corresponding values of the weight are assumed to be equidistantly spaced around zero, and are written as a *W*–dimensional vector, e.g. for a 3-state synapse ***s*** = {−1,0,1}, while for a 4-state synapse ***s*** = {−3,−1,1,3}. In numerical analysis we sometimes saw an increase in information by varying the values of the weight states, although this increase was always small. The state of any given synapse at a given time is described stochastically, by a probability vector ***π***. Each entry of ***π*** is the probability that the synapse is in that state (and hence the weight of the synapse takes the corresponding value in the weight look-up table ***s***).

Finally, we note that this setup is of course an abstraction of biological memory storage. For instance, biological coding is believed to be sparse, but the relation between our definition of *p* and actual biological coding sparsity is likely to be complicated. Our model furthermore assumes plasticity at each synapse and for every input. In some other models it has been assumed that only a subset of the inputs can cause synaptic changes [14]. Our model could in principle include this by defining null inputs that do not lead to plasticity at all. This would lead to two sparsity parameters: the proportion of inputs that induce plasticity and the proportion of plasticity-inducing inputs that lead to actual strengthening of the synapse.

#### Signal and noise

After learning, the neuron is tested on learned and novel patterns. Presentation of a learned pattern yields a signal which is on average larger than for a novel pattern. Presentation of an unlearned random pattern 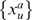 leads to a total input in the neuron 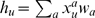. As this novel pattern is uncorrelated to the weight, it has zero mean 〈*h_u_*〉 = *n*〈*x*〉〈*w*〉 = 0, and variance
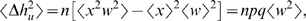
(1)where 〈*w*〉 = ***s***.***π*** ^∞^, 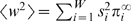, and ***π*** ^∞^ denotes the equilibrium weight distribution. The angular brackets stand for an average over many realizations of the system.

Because the synapses are assumed independent and learning is stochastic, the learning is defined by Markov transition matrices [18],[19]. The entries of these Markov matrices describe the transition probabilities between the synaptic states. If an input is high (low), the synapse is potentiated (depressed) using the Markov matrix *M* ^+^ (*M* ^−^). The distribution of the weights immediately after a high (low) input is ***π*** ^±^(*t* = 0) = *M* ^±^ ***π*** ^∞^. As subsequent uncorrelated patterns are learned, this signal decays according to ***π*** ^±^(*t*) = *M^t^* ***π*** ^±^(*t* = 0), where *t* is the discretized time elapsed since the learning of the pattern, and *M* = *pM* ^+^+*qM* ^−^ is the average update matrix. Note that the equilibrium distribution ***π*** ^∞^ is the normalized eigenvector of *M* with eigenvalue 1. When the neuron is presented with a pattern learned *t* time-steps ago, the mean signal *h* = Σ*_a_x^a^w_a_* is
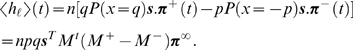
(2)This signal decays so that synapses contain most information on more recent patterns. The decay is multi-exponential, with the longest time-constant equal to the sub-dominant eigenvalue of *M*.

We define the SNR for the pattern stored *t* time-steps ago as
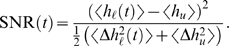
(3)For analytic work we approximate 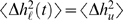, which yields with Equations 1 and 2
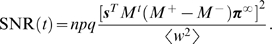
(4)


#### Information

In the testing phase we measure the mutual information in the neuron's output about whether a test pattern is learned or a novel, unlearned pattern. Given an equal likelihood of the test pattern being some learned pattern (ℓ) or an unlearned pattern (*u*), *P*(ℓ) = *P*(*u*) = 1/2, the information is given by
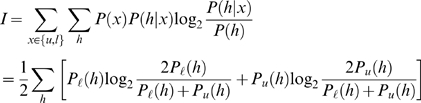
(5)where *P*_ℓ_(*h*) and *P_u_*(*h*) denote respectively the distribution of the neuron's output *h* in response to the learned and unlearned patterns. If the two output distributions are perfectly separated, the learned pattern contributes one bit of information, while total overlap implies zero information storage.

In general the full distributions *P*_ℓ_ and *P_u_* are needed to calculate the information. Unfortunately, these distributions are complicated multinomials, and can only be calculated when the number of synapses is very small (Methods). We therefore approximate the two distributions *P*_ℓ_ and *P_u_* with Gaussians, and take the variances of these distributions to be equal. An optimal threshold *θ* is imposed and the information (5) reduces to a function of the error rate *r* = *P*(*h*_ℓ_<*θ*) = *P*(*h_u_*>*θ*). This error rate is a function of the SNR, 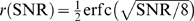. We obtain for the information
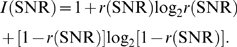
(6)Importantly, the information Equation 6 is a saturating function of the SNR. For a pattern with a very high SNR, the information approaches one bit. Meanwhile for small SNR, the information is linear in the SNR, *I*(SNR)≈SNR/(4*π*ln2).

As the patterns are independent, the total information is the sum of the information over all patterns presented during learning. We number the patterns using discrete time. The time associated with each pattern is the age of the pattern at the end of the learning phase, as measured by the number of patterns that have been subsequently presented. The total information per synapse is obtained by summing together the information of all patterns and dividing by the number of synapses, thus 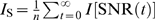. In cases in which the initial SNR is very low we approximate
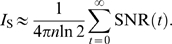
(7)In the opposite limit, when the initial SNR is very high, recent patterns contribute practically one bit of information, and we approximate as if all patterns with more than 1/2 bit actually contribute one bit, while all patterns with less information contribute zero to the information. Our numerical work shows that this is a very accurate approximation. In this limit, the storage capacity of the synapses equals the number of patterns with more than 1/2 bit of information,
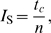
(8)where *t_c_* is implicitly defined as *I*(*t_c_*) = 1/2.

### Optimal Transfer Matrices and Information Storage

Storage capacity depends on the *W*×*W* learning matrices *M* ^+^ and *M* ^−^. To find the maximal storage capacity we need to optimize these matrices, and this optimization depends on sparseness, the number of synapses, and the number of states per synapse. Because these are Markov transition matrices, their columns need sum to one, leaving *W*(*W*−1) free variables per matrix.

#### Binary synapses, few synapses

In the case of binary synapses (W = 2) we write the learning matrices as
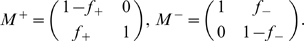
(9)We first consider the limit of few synapses, for which the initial SNR is low, and use Equation 7 to compute the information. (We keep *np*>1 and *n*≳10 to ensure that there are sufficient distinct patterns to learn.) We find
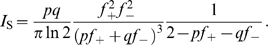
(10)The values of *f*_+_ and *f*_−_ that maximize the information depend on the sparsity *p*. There are local maxima at 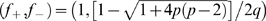 and (*f*_+_, *f*_−_) = (1,1). For 0.11<*p*<0.89, one finds that the solution (*f*_+_, *f*_−_) = (1,1) maximizes the information. In this case the synapse is modified every time-step and only stores the most recent pattern; the information stored on one pattern drops to zero as soon as the next pattern is learned. This leads to equilibrium weight distribution ***π*** ^∞^ = (*q*, *p*)*^T^* and the information is
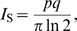
(11)which is maximal for dense coding, *I*_S_ = 0.115.

For sparser patterns *p*<0.11, the other local maximum becomes the global maximum. In particular, for small *p*, this solution is given by *f*_+_ = 1, *f*_−_≈2*p*. Thus potentiation occurs for every high input, but given a low input, depression only occurs stochasticly with probability 2*p*. Note that this is similar to the solution in [9] for binary synapses in an auto-associative network. There too, the learning rate is a factor of *p* slower when the input is negative. For this learning rule, forgetting is not instantaneous and the SNR decays exponentially with time-constant *τ* = 1/(6*p*). In the limit of very sparse patterns the associated equilibrium weight distribution is given by ***π*** ^∞^ = (2/3,1/3)*^T^*. Thus, for this regime of binary synapses and sparse patterns, at any one time one would expect to see 67% of synapses occupying the low state. This is interesting to compare to experiments in which about 80% of the synapses were found to be in the low state [7]. The information per synapse is
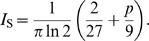
(12)There are two important observations to be made from Equations 11 and 12: 1) information remains finite at low *p*; 2) as long as the total information is small, each additional synapse contributes equally to the information.

#### Binary synapses, many synapses

We next consider the limit of many synapses, for which the initial SNR is high. With Equation 8 we find
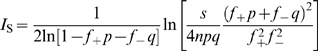
(13)where the constant *s*≈6.02 is the value of the SNR which corresponds to 1/2 bit of information. The optimal learning parameters can again be found by maximizing the information and are in this limit 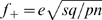 and 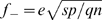, leading to equilibrium weight distribution ***π*** ^∞^ = (1/2,1/2)*^T^*. In this regime learning is stochastic, with the probability for potentiation/depression decreasing as the number of synapses increases. The intuition is that when there are many synapses, it would be wasteful for all synapses to learn about all patterns. Instead, only a small fraction of the synapses needs to store the pattern in order to have a good memory of it. The corresponding information is
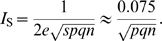
(14)Hence, as *n* becomes large, adding extra synapses no longer leads to substantial improvement in information storage capacity, but only an increase with the square root of the number of synapses. The memory decay time-constant in this case is 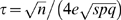.

**Figure 2:**
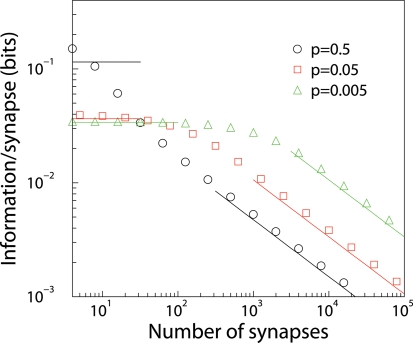
Capacity of binary synapses. Information storage capacity per synapse versus the number of synaptic inputs, for dense (*p* = 0.5), sparse (*p* = 0.05), and very sparse (*p* = 0.005) coding. Lines show analytic results, while points show numerical results. For small number of synapses, each additional synapse contributes equally to the information. However, for many synapses, information per synapse decreases as 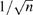.

To verify the above results, and to examine the information between the large and small *n* limits, we numerically maximized the information by searching the space of possible learning matrices (Methods). This means that for each data point we optimized the parameters *f*_+_ and *f*_−_. We find there is a smooth interpolation between the two limiting cases, and good match with the theory. For given sparsity, there is a critical number of synapses beyond which addition of further synapses does not substantially improve information capacity, Figure 2. This critical number is the point at which the direct proportionality of the information to the SNR Equation 7, breaks down. That is, the *n* for which the initial SNR becomes of order 1. For dense patterns, this occurs for just a few synapses, while for sparse patterns this number is proportional to *p* ^−1^.

In terms of *total* information, this result means there is linear growth for small number of synapses, but beyond the critical number addition of further synapses only leads to an increase with the square root of the number of synapses, a rather less substantial growth.

#### Comparison with Willshaw net

We compare the storage capacity found here with that of a Willshaw net [1]. This is of interest as this also uses binary synapses, although in a non-stochastic manner, and has a high capacity. In Willshaw's model, all synapses initially occupy a silent (*w* = 0) state, and learning consists solely of potentiation to an active (*w* = 1) state when a high input is presented. Each input *x* takes the value 0 (off) or 1 (on), and each pattern contains a fixed number, *np*, of positive inputs. As more patterns are presented, more synapses move to the active state, and eventually all memories are lost. However, when only a finite, optimal number of patterns are presented, this performs well.

Since a learned pattern definitely gives the signal *h* = *np*, the threshold for recognizing a pattern as “learned” is set to *h* = *np*. When an unlearned pattern is presented, there is still a chance that the response will be “learned”. When *m* patterns have been presented, the chance that a given synapse is still in the silent state is *q^m^*. Hence the probability of an unlearned pattern being falsely recognized as “learned” is *ε* = (1−*q^m^*)*^np^*. This is the only source of error. The information stored on any one pattern is found from Equation 5, restricted to binary output:
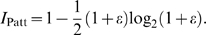
(15)The total information per synapse *I*_S_ = (*m*/*n*)*I*_Patt_. Given the number of synapses, and the sparsity, one can optimize the information with respect to the number of patterns. In the limit of few synapses, and sparse patterns, one can achieve *I*_S_ = 0.11 bits, which is several times higher than the storage we obtain for our model when coding is sparse. However, as the number of synapses increases, storage decays with *n* ^−1^, which is much faster than the *n* ^−1/2^ decay found here. (Aside: Willshaw obtains a maximum capacity of *I*_S_ = 0.69 bits within his framework [1],[20]. This is for an associative memory task, and a different information measure from that considered here. There the expected number, *E*, of errors in the output is calculated as a function of the number of stored associations. The number, *m*, of associations that are then presented is that for which *E* = 1. The information stored is defined as the total information content of the *m* output patterns presented.)

#### Multi-state synapses

Next, we examine whether storage capacity increases as the number of synaptic states increases. Even under small or large *n* approximations, the information obtained from Equation 4 is in general a very complicated function of the learning parameters, due to the complexity of the invariant eigenvector ***π*** ^∞^ of a general Markov matrix *M*. Thus optimal learning must be found numerically by explicitly varying all matrix elements; this must be restricted to synapses with just a few states (up to 8). For large *n* we find that the optimal transfer matrix is band diagonal, meaning the only transitions are one-step potentiation and depression. Moreover, we find that for fixed number of synaptic states, the (optimized) information behaves similar to that of binary synapses.

In the dense (*p* = 1/2) case, we find that the optimal learning rules balance potentiation and depression, by satisfying (*M* ^+^)*_ij_* = (*M* ^−^)*_W_*_+1−*j*,*W*+1−*i*_. In the limit of many synapses, the optimal learning rule takes a simple form
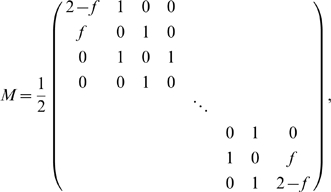
(16)with 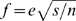.

Perhaps one would expect optimal storage if, in equilibrium, synapses were uniformly distributed, thus making equal use of all the states. However, the equilibrium weight distribution is peaked at both ends, and low and flat in the middle, ***π*** ^∞^∝(1,*f*,*f*,…,*f*,1)*^T^*. The associated information is
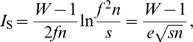
(17)and the corresponding time-constant for the SNR is given by 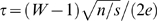. Importantly, the information grows linearly with the number of synaptic states. However, validity of these results requires *fW* to be small to enable series expansion in *f*, i.e. information is linear in *W* if 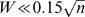.

**Figure 3:**
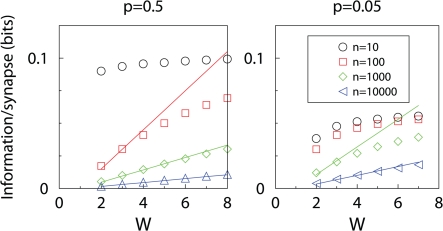
Capacity of multi-state synapses. Information storage capacity per synapse versus the number *W* of synaptic states, for dense (*p* = 0.5) and sparse (*p* = 0.05) coding. Lines show analytic results (when available), whilst points show numerical results. When the neuron has many synapses, the storage capacity initially increases with the number of synaptic states, but eventually saturates.

In the sparse case there seems to be no simple optimal transfer matrix, even in the large *n* limit. However, we can infer a formula for *I*_S_ from our analytic and numerical results. A formula consistent with the binary synapse information Equation 14, as well as the case of dense patterns, Equation 17 is
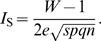
(18)Assuming that this formula, as for the binary synapse, is the leading term in a series expansion in the parameters 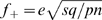 and 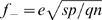, and that we need *Wf*_+_ and *Wf*_−_ small for it to be accurate, Equation 18 is valid when 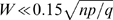. We have confirmed from simulations that this formula is a good fit for a wide range of parameters, Figure 3.

For large *W*, or equivalently small *n*, the capacity saturates and becomes independent of *W*, see Figure 3. This is also observed with a number of different (sub-optimal) learning rules studied in [18]. These learning rules had the property that the product of initial SNR and the time-constant *τ* of SNR decay is independent of *W*. See Table 1 in [18] for this remarkable identity, noting that the SNR there equals its square root here, and that *α* = 1/*W*. For large *W* the initial SNR is small, and hence the information can be approximated as *I*∼Σ*_t_*SNR(0)exp(−*t*/*τ*)≈*τ* SNR(0). Also for the optimal learning rule studied here the information becomes independent of *W*, Figure 3.

#### Hard-bound learning rules

Finally we study, for large *n*, the performance of a simple “hard-bound” learning rule, i.e. a learning rule that yields a uniform equilibrium weight distribution. Under this rule, whose SNR dynamics were previously studied in [18], a positive (negative) input gives one-step potentiation (depression) with probability *f*_+_ (*f*_−_). I.e. 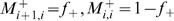, but 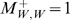. For *W*≥4 the optimal probabilities satisfy 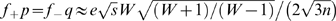 [18], for which
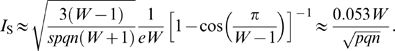
(19)Here the latter approximation is for large *W*. The time-constant of the SNR decay is 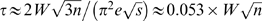. This sub-optimal learning rule gives an information capacity of the same functional form as the optimal learning rule, but performs only 70% as well.

Given that simple stochastic learning performs almost as well as the optimal learning rule, we wondered how well a simple deterministic learning rule performs in comparison. In that case, synapses are always potentiated or depressed, there is no stochastic element, i.e. *f*_+_ = *f*_−_ = 1. One finds
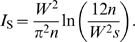
(20)The memory decay time here is *τ* = *W* ^2^/*π* ^2^. Although the information grows faster with *W*, the 1/*n* behavior means this performs much worse than optimal stochastic learning for any reasonable number of synapses. Interestingly, 1/*n* is the same decay as for the Willshaw net, suggesting that this is a general feature of deterministic learning rules.

#### Comparison to continuous, unbounded synapses

The above results raise the question whether binary synapses are much worse than continuous synapses. It is interesting to note that even continuous, unbounded synapses can store only a limited amount of information. We consider a setup analogous to that of Dayan and Willshaw [2]. Prior to learning, all weights are set to zero. Learning involves potentiation by a fixed amount when a positive input is presented, and depression by a fixed amount when a negative input is presented. With *m* patterns learned, the mean and variance of the output for an unlearned pattern are respectively 〈*h_u_*〉 = 0 and 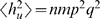, while for a learned pattern, 〈*h*_ℓ_〉 = *npq*. Hence SNR = *n*/*m* for all patterns. The information is maximal at *I*_S_≈0.11 when *m*≫*n*≫1. This result indicates that under the right conditions the capacity of binary synapses indeed approaches that of continuous unbounded synapses. Note that in this model *I*_S_ is independent of *n* for large *n*. This is consistent with the results above for bounded synapses: in the limit *W*→∞ one necessarily enters the regime in which *I*_S_ is independent of *n*.

#### Associative learning

**Figure 4:**
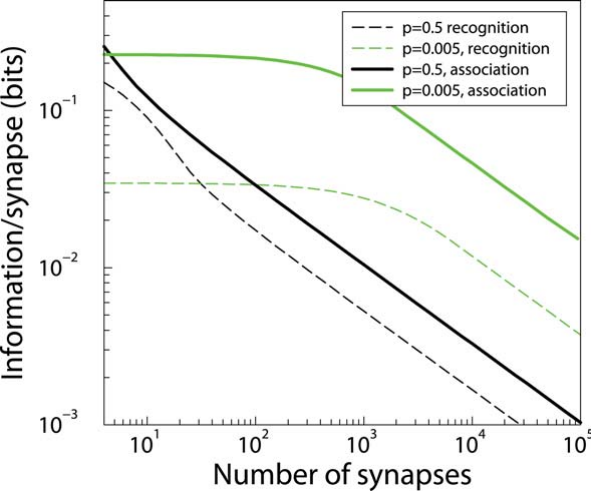
The memory information capacity of a neuron with binary synapses that has been trained on an association task. The capacities for the recognition task (Figure 2) are redrawn for comparison (dashed lines). The capacities for association (solid lines) are higher but follow the same trend.

In all the above the neuron's task was to correctly recognize patterns that were learned before. We wondered if our results generalize to a case in which the neuron has to associate one half of the patterns to a low output and the other half of the patterns to a high output. This is a supervised learning paradigm which is specified by defining what happens when the input is high/low and the desired output is high/low. In other words, there are four learning matrices [19]. The analysis of this case is therefore more complicated. The result of simulations that optimize these matrices is shown in Figure 4. The information storage is higher than for the task above, by about a factor 2 for dense patterns, and a factor 4 for sparse patterns. However, the shape of the matrices and the qualitative dependence on the number of synapses is the same, demonstrating that qualitatively our conclusions carry over to other learning paradigms as well.

## Discussion

We have studied pattern storage using discrete, bounded synapses. Learning rules for these synapses can be defined by stochastic transition matrices [18],[19]. In this setup an SNR based analysis provides two contradictory measures of performance: the quality of learning (the initial SNR), and the rate of forgetting [18]. With our single measure of storage capacity based on Shannon information, learning rules can be optimized. The optimal learning rule depends on the number of synapses *n* and the coding sparseness *p*, as well as on the number of states *W*. Our analysis was restricted to about 8 states per synapse, although we have no reason to believe that extrapolation to larger numbers would not hold.

Given optimal learning we find two regimes for the information storage capacity: 1. When the number of synapses is small, information per synapse is constant and approximately independent of the number of synaptic states. 2. When the number of synapses is large, capacity per synapse increases linearly with *W* but decreases as 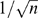. The critical *n* that separates the two regimes is dependent on sparseness and the number of weight states. The optimal learning rule for regime 2 has band-diagonal transition matrices, and in the dense case (*p* = 1/2), these take a particularly simple form, see Equation 16. Capacity of order 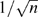 in the large *n* limit has been reached in other studies of bounded synapses [10],[21], but has not been exceeded to our knowledge. It remains a challenge to construct a model that does better than this.

The implications for biology depend on the precise nature of single neuron computation. If a neuron can only compute the sum of all its inputs then we might conclude the following. As synapses are metabolically expensive [22], biology should choose parameters such that the number of synapses per neuron does not exceed the critical number much. Although there are currently no accurate biological estimates for either the number of weight states, or the sparsity, for binary synapses with *p* = 0.005, the critical number of synapses is close to the number of synapses (∼10,000) per neuron in the hippocampus (see Figure 2). However, if the neurons can do compartmentalized processing so that the dendrite is the unit of computation [23], then one could think of this model as representing a single dendrite, and we could conclude that the number of synapses per dendrite might be optimized for information storage capacity. For binary synapses with *p* = 0.005 choosing the number of synapses to be several hundred is also close to optimal.

Furthermore, our results predict that when synapses are binary, coding is sparse, and learning is optimized, that at equilibrium about 67% of synapses should occupy the low state. This is not far off the experimental figure of 80% [7].

We have directly compared discrete to continuous synapses. For few synapses and dense coding, binary synapses can store up to 0.11 bits of information, which is comparable to the maximal capacity of continuous synapses. However, for sparse coding and many synapses per neuron, the capacity of binary synapses is reduced. Hence, if one considered only information storage, one would conclude that, unsurprisingly, unbounded synapses perform better than binary synapses. However, in unbounded synapses, weight decay mechanisms must be introduced to prevent runaway, so the information storage capacity is necessarily reduced in on-line learning [16],[17]. In contrast, for bounded, discrete synapses with ongoing potentiation and depression, such as those considered here, old memories undergo “graceful decay” as they are automatically overwritten by new memories [8],[9],[12],[13],[15]. Thus discrete, bounded synapses allow for realistic learning with a good capacity.

Finally, it is worth noting that although using Shannon information is a principled way to measure storage, it is unclear whether for all biological scenarios it is the best measure of performance, c.f. [24]. The information can be higher when storing very many memories with a very low SNR, than when storing just a few patterns very well. This might be undesirable in some biological cases. However, if many neurons work in parallel on the same task, it is likely that all information contributes to performance, and thus the total bits per synapse is a useful measure.

## Methods

To obtain the information capacity numerically, we used Matlab and implemented the following process. For a given number of synaptic states, number of synapses and sparsity, we used Matlab's fminsearchbnd to search through the parameter space of all possible transfer matrices *M* ^+^ and *M* ^−^. That is, all matrix elements were constrained to take values between 0 and 1, and all columns were required to sum to 1. For each set of transfer matrices we first obtained the equilibrium weight distribution ***π*** ^∞^ as the eigenvector with eigenvalue 1 of the matrix *M*. Then we computed the means and variances of the output for learned and unlearned patterns from Equations 2 and 1, and further used that 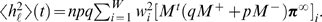. Equations 6 and 3 then gave the information stored about the pattern presented at each time-step. To calculate the total information, this was summed over sufficient time-steps.

In particular, in the case of many weight states (large *W*) and sparse patterns, the maximization would sometimes get stuck in local maxima. In those cases we did multiple (up to 50) restarts to make sure that the solution found was truly optimal.

Our results can also be compared to the so-called cascade model, which was recently proposed to have high SNR and slow memory decay [10]. In order to compare the cascade model to our results, we created a six-state cascade model using learning matrices that only had transitions according to the state diagram in [10]. These transition rates were then optimized. We found that the information capacity of the optimized cascade model was always larger than a two-state model, but always lower than our six state model with transfer matrix Equation 16. Only when the number of synapses was small (and the information became equal to the integral over the SNR), did the two-state, six-state and cascade models give identical performance. For a higher number of states the results could be different, but this study suggests that, at least for a small number of states, the cascade model is sub-optimal with respect to Shannon information capacity.

Finally, we explored how well the Gaussian approximation worked. We calculated the full multinomial distribution of the total input *h* and applied an optimal threshold. Because of a combinatorial explosion, this was only feasible for up to 100 synapses. When the information was maximized this way, the information increased to about 0.3 for *n* = 1 binary synapses storing dense patterns, but for more than *n* = 10 synapses the results were indistinguishable from the presented theory.
